# The cost-effectiveness of adding an ultrasound corticosteroid and local anaesthetic injection to advice and education for hip osteoarthritis

**DOI:** 10.1093/rheumatology/kead659

**Published:** 2023-12-12

**Authors:** Jesse Kigozi, Raymond Oppong, Zoe Paskins, Kieran Bromley, Martyn Lewis, Gemma Hughes, Emily Hughes, Susie Hennings, Andrea Cherrington, Alison Hall, Melanie A Holden, Kay Stevenson, Ajit Menon, Philip Roberts, George Peat, Clare Jinks, Nadine E Foster, Christian D Mallen, Edward Roddy

**Affiliations:** Health Economics Unit, Institute of Applied Health Research, University of Birmingham, Birmingham, UK; Health Economics Unit, Institute of Applied Health Research, University of Birmingham, Birmingham, UK; School of Medicine, Keele University, Keele, UK; Haywood Academic Rheumatology Centre, Midlands Partnership University NHS Foundation Trust, Stoke-on-Trent, UK; Keele Clinical Trials Unit, Keele University, Keele, UK; Keele Clinical Trials Unit, Keele University, Keele, UK; Keele Clinical Trials Unit, Keele University, Keele, UK; Keele Clinical Trials Unit, Keele University, Keele, UK; Keele Clinical Trials Unit, Keele University, Keele, UK; Keele Clinical Trials Unit, Keele University, Keele, UK; School of Medicine, Keele University, Keele, UK; School of Medicine, Keele University, Keele, UK; Haywood Academic Rheumatology Centre, Midlands Partnership University NHS Foundation Trust, Stoke-on-Trent, UK; Haywood Academic Rheumatology Centre, Midlands Partnership University NHS Foundation Trust, Stoke-on-Trent, UK; University Hospitals North Midlands, Stoke-on-Trent, UK; School of Medicine, Keele University, Keele, UK; Centre for Applied Health & Social Care Research (CARe), Sheffield Hallam University, Sheffield, UK; School of Medicine, Keele University, Keele, UK; School of Medicine, Keele University, Keele, UK; Surgical Treatment And Rehabilitation Service (STARS), Research and Education Alliance, The University of Queensland and Metro North Health, Brisbane, QLD, Australia; School of Medicine, Keele University, Keele, UK; Haywood Academic Rheumatology Centre, Midlands Partnership University NHS Foundation Trust, Stoke-on-Trent, UK; School of Medicine, Keele University, Keele, UK; Haywood Academic Rheumatology Centre, Midlands Partnership University NHS Foundation Trust, Stoke-on-Trent, UK

**Keywords:** economic evaluation, cost-effectiveness, cost–utility, hip osteoarthritis, corticosteroid injection

## Abstract

**Objectives:**

Evidence for the comparative cost-effectiveness of intra-articular corticosteroid injection in people with hip osteoarthritis (OA) remains unclear. This study investigated the cost-effectiveness of best current treatment (BCT), comprising advice and education, with BCT plus a single ultrasound-guided intra-articular hip injection of 40 mg triamcinolone acetonide and 4 ml 1% lidocaine hydrochloride (BCT+US-T).

**Methods:**

A trial-based cost–utility analysis of BCT+US-T compared with BCT was undertaken over 6 months. Patient-level cost data were obtained, and effectiveness was measured in terms of quality-adjusted life years (QALYs), allowing the calculation of cost per QALY gained from a UK National Health Service (NHS) perspective.

**Results:**

BCT+US-T was associated with lower mean NHS costs (BCT+US-T minus BCT: −£161.6; 95% CI: −£583.95, £54.18) and small but significantly higher mean QALYs than BCT alone over 6 months (BCT+US-T minus BCT: 0.0487; 95% CI: 0.0091, 0.0886). In the base case, BCT+US-T was the most cost-effective and dominated BCT alone. Differences in total costs were driven by number of visits to NHS consultants, private physiotherapists and chiropractors, and hip surgery, which were more common with BCT alone than BCT+US-T.

**Conclusion:**

Intra-articular corticosteroid injection plus BCT (BCT+US-T) for patients with hip OA results in lower costs and better outcomes, and is highly cost-effective, compared with BCT alone.

**Trial registration:**

EudraCT: 2014-003412-37 (8 August 2015) and registered with Current Controlled Trials: ISRCTN 50550256 (28 July 2015).

**Trial protocol:**

Full details of the trial protocol can be found in the Supplementary Appendix, available with the full text of this article at https://bmcmusculoskeletdisord.biomedcentral.com/articles/10.1186/s12891-018-2153-0, doi: doi.org/10.1186/s12891-018-2153-0.

Rheumatology key messages

- Economic evidence supporting the use of intra-articular corticosteroid injection for hip osteoarthritis is currently lacking.
- Ultrasound-guided intra-articular hip injection combined with advice and education is cost-effective in hip OA patients.
- Findings provide health economics evidence to inform guidelines and offer important choice to patients.

## Introduction

Hip osteoarthritis (OA) is one of the most common musculoskeletal diseases and results in global disability, functional loss and impaired quality of life [1, 2]. Between 10% and 18% of those aged over 60 years are affected, rising to one in three over the age of 80 years [3]. Hip OA is a significant contributor to the economic burden on society, with the cost of total hip replacement surgery in the UK exceeding £500 million in 2019 [4].

Given the economic impact of hip OA, it is important to explore cost-effective options for improving patient outcomes of pain, function and quality of life. National Institute for Health and Care Excellence (NICE) guidance recommends combining non-pharmacological and pharmacological treatments, with education, exercise and weight reduction being core treatments [5]. Analgesic options include paracetamol, with intra-articular corticosteroid injections to be considered when other pharmacological treatments are ineffective or unsuitable. However, the economic evidence supporting the use of intra-articular hip corticosteroid injection remains unclear and none of the published randomized controlled trials (RCTs) have examined cost-effectiveness [5, 6].

We report the health economic evaluation conducted alongside the Hip Injection Trial (HIT) [7, 8]. The clinical results of the HIT trial found that an ultrasound-guided intra-articular hip injection of triamcinolone acetonide and lidocaine hydrochloride combined with best current treatment led to greater pain reduction and improvement in function over a 6-month period in adults with hip OA [7]. The aim of the economic evaluation is to determine the cost-effectiveness of an ultrasound-guided intra-articular hip injection (USGI) of 40 mg triamcinolone acetonide and 4 ml 1% lidocaine hydrochloride combined with best current treatment (BCT+US-T) in patients with hip OA, compared with best current treatment (BCT) alone.

## Methods

### Trial design

The HIT trial protocol and comparative clinical effectiveness findings have been reported in detail elsewhere [7, 8]. The trial was a pragmatic, three-arm parallel-group, single-blind RCT that recruited patients between 18 January 2016 and 21 May 2018 within the National Health Service (NHS) in England. Patients aged ≥40 years with moderate–severe pain attributable to hip OA present for 6 weeks or more and occurring on most days in the last month were eligible and were invited to participate in the trial. Participants were recruited following referral from primary care to orthopaedics, rheumatology or two musculoskeletal interface services (MIS), and directly from general practices.

### Ethics

This study was approved by the National Research Ethics Service Committee North West (UK) Central (15/NW/0546) and Medicines and Healthcare Products Regulatory Agency (2014-003412-37). Participants gave full informed written consent before study procedures.

### Interventions

All participants in the trial received BCT at baseline. BCT comprised written information (Versus Arthritis Osteoarthritis leaflet), a bespoke leaflet on exercise and functional activities (https://doi.org/10.21252/1w07-2214), and advice and information about weight loss, exercise, footwear, walking aids and pain management in one session with an assessing clinician. Participants were randomized 1:1:1 to one of three treatment arms: (i) BCT alone, (ii) BCT plus USGI of triamcinolone acetonide and 1% lidocaine hydrochloride (BCT+US-T), or (iii) BCT plus USGI of 1% lidocaine hydrochloride only—using random permuted blocks of three and six, via Keele CTU’s secure web-based randomization service. Participants randomized to BCT did not receive any further treatment in the trial. Those randomized to BCT+US-T received one USGI of triamcinolone acetonide 40 mg/ml sterile, aqueous solution (Bristol Myers Squibb, Dublin, Ireland) and 4 ml 1% lidocaine hydrochloride (Hameln Pharmaceuticals, Gloucester, UK). The injection was delivered by a different clinician (trained rheumatologist, extended scope physiotherapist or sonographer) from the one who assessed and delivered BCT, but was given on the same day of assessment. More comprehensive details on the trial design and interventions are reported elsewhere [8]. This study reports the health economic analysis of BCT+US-T compared with BCT within a cost–utility analysis framework. In line with the study protocol and health economics analysis plan, cost-effectiveness of BCT plus USGI of 1% lidocaine hydrochloride only was not examined, as USGI of 1% lidocaine hydrochloride only is not undertaken as a treatment in routine clinical practice.

### Economic study design: overview

The trial-based evaluation was a cost–utility analysis, adopting an NHS perspective, to determine the cost-effectiveness of BCT+US-T compared with BCT alone for participants with hip OA over a period of 6 months with quality-adjusted life years (QALYs) as the health outcome. QALYs account for quality of life and survival, and in this study we sought potential quality of life gains from reduction in hip pain intensity from the two interventions. The base case analysis was based on the intention-to-treat population and conducted from the perspective of UK National Health Service and Personal Social Services (NHS/PSS). The time horizon for follow-up was 6 months and costs were not discounted as the trial follow-up of each participant was limited to 6 months. Results were reported in according with the Consolidated Health Economic Evaluation Reporting Standards (CHEERS) [9].

### Health outcomes

Health-related quality of life was participant-reported and collected at 2 weeks and 2, 4 and 6 months post-randomization using the Euroqol’s EQ-5D-5L questionnaire, a generic instrument for measuring and valuing health-related quality of life [10]. In line with current guidelines, responses from each time point were converted into index scores [ranging from −0.594 (the worst health state) to 1.000 (full health), with 0 equivalent to death]. The mapping function developed by the Decision Support Unit, using the ‘EEPRU dataset’, was used for mapping the data from the EQ-5D-5L to the EQ-5D-3L [11]. Quality-adjusted life-years (QALYs) were then generated for each participant using the area under the baseline-adjusted utility curve, assuming linear interpolation across follow-up time points [12]. To avoid bias, adjustment for differences between the two arms in baseline EQ-5D-5L utility scores was undertaken using regression-based adjustments [13]. EQ-5D-5L values and QALYs generated over 6 months were then reported by treatment group and presented as means and standard deviations. We used EQ-5D-5L scores to calculate QALYs.

### Resource use and cost analysis

In the base-case analysis, participant-specific resource use data were collected from self-report postal questionnaires administered at 6 months. NHS resource use data included primary and community care contacts (face-to-face general practice doctor, practice nurse, community therapy, and other primary contacts), hospital-based services (e.g. consultants, outpatient appointments, physiotherapy, inpatient admissions, diagnostic tests, injections, scans and surgical procedures) and prescribed medication. Non-NHS (healthcare) costs were obtained by asking participants about their use of other private health care and purchase of over-the-counter medicines, appliances and devices and treatments. In order to assess the broader economic consequences of the interventions beyond healthcare resources, self-reported data on occupation and time taken off work owing to their hip OA over the 6-month period were also collected.

Information on resource use was also collected within the trial to estimate the costs of delivering the intra-articular corticosteroid injection for each participant. The costs of the intervention included healthcare personnel (rheumatologist, physiotherapist, consultant sonographer) and the ultrasound machine. The ultrasound machine costs were annuitized over an expected life span of 10 years. The cost of the advice and information booklet was provided for all participants and was therefore not included in the final analysis as it would not contribute to the incremental cost-analysis between the groups. Health resource use information was obtained from the self-reported questionnaires at 6 months and these were valued with unit cost data from standard sources, including the Unit Costs of Health and Social Care [14], NHS reference costs [15], and the British National Formulary [16]. Productivity costs were estimated by multiplying productivity loss outputs with the average wage rate identified from annual earnings data [17]. The analysis used the human capital approach [18]. Supplementary Tables S1 and S2, available at *Rheumatology* online, present details of the unit costs assigned to health care resource use data and lost productivity.

### Statistical analysis

Analysis was conducted by intention-to-treat following a pre-agreed health economics analysis plan. The main comparison was between BCT+US-T *vs* BCT alone to estimate mean incremental QALYs and hip-pain related healthcare costs using a within-trial analysis. Missing EQ-5D-5L and total cost data were imputed using multiple imputation by chained equation and predictive mean matching, assuming that values are missing at random to address issues with incomplete data [19, 20]. Appropriateness of this missing at random assumption was based on comparisons of the characteristics of patients with and without missing costs and quality of life data at the follow-up time points. The imputed dataset was used as the primary dataset for all analyses. The imputation model included 25 imputed datasets, and Rubin’s rule was used to combine the imputed datasets into one final imputed variable [21]. The primary outcome of interest was the cost per additional QALY gained. In order to account for uncertainty, bootstrapping techniques were used to derive 5000 estimates of mean differential cost and QALY scores and presented on a cost-effectiveness plane [22]. The analysis was based on seemingly unrelated regression techniques for simultaneous estimation of cost and health outcomes. A cost-effectiveness acceptability curve was estimated showing the probability that the treatment was cost-effective across a range of possible values of willingness to pay for an additional QALY [23]. Total health care costs over the study period were calculated by multiplying the resource items used by the respective unit cost and summing over all items. The analytic comparison across arms focused on the joint estimation of incremental costs and incremental QALYs (with increments calculated as the BCT+US-T minus BCT). Bootstrapped 95% confidence intervals for the between-group differences in QALYs and cost estimates were also reported. All analyses were performed using Stata V.16 (StataCorp, College Station, TX, USA) [24].

Sensitivity analyses were performed to (i) estimate the cost-effectiveness from a healthcare perspective, including private and patient related costs, (ii) consider a broader societal perspective, including patient specific productivity costs in addition to healthcare costs (using the human capital approach to value productivity losses due to self-reported work absence over the 6-month period, stratified by gender and full-time/part-time work status average wage rates), (iii) evaluate cost–utility by use of complete-case analysis, and (iv) undertake a *post hoc* cost–utility analysis excluding surgery costs in the BCT-alone group. Costs or health outcomes were not discounted because of the 6-month follow-up. Responses from each time point were converted into index scores using the interim cross-walk value set for mapping EQ-5D-5L data to the EQ-5D-3L [25].

## Results

### Response rates and data completion

A total of 133 (BCT alone *n* = 67, BCT+US-T *n* = 66) people with hip OA formed the dataset for the analysis. All base-case analysis reflects the imputed dataset unless stated otherwise. Cost estimates were available for 111 (84%) responders at 6 months. Complete EQ-5D-5L outcome data at all time points were available for 97 participants (73% of the total sample). At each of the time points, complete EQ-5D data were available for 98% (baseline), 93% (2 weeks), 89% (2 months), 86% (4 months) and 83% (6 months). Overall, baseline characteristics where similar between the two groups but the BCT-alone group had more women and shorter pain duration, while participants in the BCT+US-T group were more likely to have a paid job and less likely to have comorbidities.

### Health outcomes

Mean EQ-5D-5L scores at baseline and follow-up time points and mean QALYs are shown in Table 1. Health-related quality of life in the BCT+US-T group was higher at 6 months compared with baseline. In the BCT-alone group, health-related quality of life (HRQOL) was lower at 6 months’ follow-up compared with baseline. The adjusted and imputed mean QALYs over 6 months were higher for the BCT+US-T group than for the BCT group (QALY difference 0.0487; 95% CI: 0.0091, 0.0886).

**Table 1.** Descriptive and incremental health outcomes over 6 months for the base-case analysis and the complete case analyses

	BCT	BCT+US-T	Difference^a^ (95% CI)
Primary (imputed) EQ-5D analysis			
*n*	67	66	
Baseline EQ-5D	0.4705 (0.2384)	0.4520 (0.2594)	−0.0184 (−0.1045, 0.0636)
2 weeks EQ-5D	0.4472 (0.2977)	0.6356 (0.2575)	0.1883 (0.09479, 0.2858)
2-month EQ-5D	0.4225 (0.2973)	0.5603 (0.2847)	0.1378 (0.0424, 0.2350))
4-month EQ-5D	0.4441 (0.2961)	0.5515 (0.2753)	0.1073 (0.0064, 0.2030)
6-month EQ-5D	0.4660 (0.2692)	0.4598 (0.2851)	−0.0061 (−0.0995, 0.0861))
Unadjusted total QALYs	0.2045 (0.1221)	0.2532 (0.1165)	0.0487 (0.0091, 0.0886)
Adjusted total QALYs^b^	—	—	0.05572 (0.0308, 0.0806)
Complete-case analysis			
* n*	47	50	
Unadjusted total QALYs	0.2098 (0.1297	0.2532 (0.1165)	0.0564 (0.0084, 0.1070)
Adjusted total QALYs^c^	—	—	0. 0562 (0.0258, 0.0864)

Values are mean (s.d.) scores unless stated otherwise.

a Difference=BCT+US-T minus BCT. Reported CIs were generated using regression methods.

b Adjusted for baseline utility. BCT: best current treatment; BCT+US-T: BCT plus US-guided injection of triamcinolone and lidocaine; QALY: quality-adjusted life year; EQ-5D: EuroQol-5D.

### Resource use and costs

Table 2 shows the disaggregated details of mean resource for participants with complete resource use data. Over 6 months, small differences in the uptake of primary care and secondary care NHS services were found between the two groups with the exception of the number of visits to NHS consultants, visits to private physiotherapist, and NHS hip-related surgeries. The number of NHS consultant and private physiotherapist visits were higher in the BCT-alone group, and two participants reported having hip-related surgery (largest cost driver) in the BCT group compared with none in the other group.

**Table 2.** Hip OA-related healthcare resource use per patient, by treatment group, for patients providing utilisation data at 6 months (*n* = 111)

Health care resource	BCT (*n* = 53)	BCT+US-T (*n* = 58)	Difference (95% CI)^a^
Primary care general practitioner	0.85 (1.38)	0.78 (1.11)	−0.07 (−0.56, 0.38)
Primary care nurse	0.15 (0.49)	0.17 (0.65)	0.02 (−0.17, 0.26)
Prescriptions	2.38 (3.63)	1.89 (3.17)	−0.48 (−1.73, 0.73)
NHS consultant	0.40 (1.74)	0.26 (0.55)	−0.14 (−0.78, 0.22)
Private consultant	0.00	0.02 (0.13)	0.02 (0, 0.07)
NHS physiotherapist	0.30 (0.89)	0.26 (0.74)	−0.04 (−0.38, 0.25)
Private physiotherapist	0.42 (1.77)	0.09 (0.66)	−0.33 (−0.96, 0.08)
Private chiropractor	0.32 (1.59)	0.00 (0.00)	−0.32 (−0.85, 0)
Private acupuncturist	0.00 (0.00)	0.21 (1.57)	0.21 (0, 0.75)
NHS X-rays	0.17 (0.51)	0.24 (1.09)	0.07 (−0.18, 0.46)
NHS US scans	0.04 (0.19)	0.09 (0.54)	0.05 (−0.05, 0.27)
NHS CT scans	0.00 (0.00)	0.02 (0.13)	0.02 (0, 0.67)
NHS blood tests	0.11 (0.51)	0.31 (1.37)	0.19 (−0.08, 0.66)
NHS MRI investigations	0.02 (0.14)	0.05 (0.29)	0.03 (−0.03, 0.13)
Resource use other			
NHS hip-related surgery, *n* (%)	2 (4)	0	—

Values are mean (s.d.) unless stated otherwise.

a Between-group difference in mean scores (BCT+US-T minus BCT), USGI ultrasound-guided intra-articular hip injection of 40 mg triamcinolone acetonide and 4 ml 1% lidocaine hydrochloride. BCT: best current treatment; BCT+US-T: BCT plus US-guided injection of triamcinolone and lidocaine.

Less than half the patients in the trial were in paid employment at 6-month follow-up: 20 (38%) in the BCT group and 31 (54%) in the BCT+US-T group. Data on employment and time-off work are reported in Table 3. Of those who reported being in employment, 4 (8%) patients in the BCT group reported time off paid work because of hip pain, compared with 8 (14%) in the BCT+US-T. During the 6-month follow-up, the mean number of days off work was higher in the BCT-alone group (6.25 days) than in the BCT+US-T group (1.39 days). This translated into higher productivity costs in the BCT group (£599.7) compared with BCT+US-T group (£149.5). Overall, participants reporting time off work in the BCT-alone group were associated with more days off work than respondents in the BCT+US-T group (Table 3).

**Table 3.** Description of work-related outcomes for participants in paid employment by treatment group

Work-related outcomes^a^	BCT	BCT+US-T
Baseline: working in paid employment, *n* (%)	25/67 (37)	37/66 (56)
Baseline: reported time off work during the last 6 months, *n* (%)	3 (5)	8 (12)
Baseline: paid employment reported time off work during the last 6 months, *n* (%)	3 (12)	8 (21)
Working in paid employment at 6- months follow-up, *n* (%)	20/53 (38)	31/58 (54)
Number of patients reporting time-off work at 6 months, *n* (%)	4 (8)	8 (14)
Performance at work at 6 months, mean (s.d.)^b^	4.40 (3.29)	4.12 (2.68)
Difference, mean (95% CI)^b^	−0.28 (−2.0, 1.40)	
Days off-work at 6 months, mean (s.d.)	6.25 (18.69)	1.39 (3.99)
Difference, mean (95% CI)	−4.86 (−15.03,1.12)	
Productivity costs, mean (s.d.)^c^	599.69 (2141.68)	149.53 (447.84)
Difference, mean (95% CI)	−450.16 (−1647.74, 131.61)	

a The evaluation of work-related outcomes and the estimation of indirect costs focused on the subsample of respondents in paid employment at 6 months (51/113).

b Mean performance at work on a scale of 0–10 where 0 indicates work performance not affected, 10 is for most affected.

c Productivity costs obtained from days off-work at 6 months. BCT best current treatment; BCT+US-T: BCT plus US-guided injection of triamcinolone and lidocaine.

Table 4 shows the disaggregated mean (s.d.) NHS cost per patient for each intervention and the total cost estimates for the base-case imputed analysis. The mean total costs per patient were £327.5 (1331.9) for BCT alone compared with £165.8 (212.7) for BCT+US-T. These costs reflect the higher resource use in the BCT-alone group attributable to NHS consultant visits and NHS hip-related surgeries.

**Table 4.** Hip OA-related healthcare costs (£) per patient, by treatment group, for patients providing utilisation data at 6 months (*n* = 111)

Health care resource	BCT (*n* = 53)	BCT+US-T (*n* = 58)	Difference (95% CI)^a^
Intervention cost per patient^b^	—	33.54	33.54
General practitioner visit	26.32 (42.73)	24.05 (34.38)	−2.26 (−17.60, 12.68)
Nurse visit	5.43 (17.86)	6.21 (23.49)	0.77 (−6.35, 9.16)
Primary care other			
Prescriptions	17.03 (37.91)	10.94 (23.71)	−6.09 (−19.60, 4.28)
NHS consultant visit	71.72 (314.18)	46.81 (99.19)	−24.91 (−158.88, 37.11)
Private consultant visit	0.00	3.12 (23.76)	3.12 (0,12.48)
NHS physiotherapist	16.60 (48.93)	14.22 (40.64)	−2.38 (−19.53, 13.48)
Private physiotherapist	22.83 (97.33)	4.74 (36.11)	−18.09 (−53.65, 4.58)
Private chiropractor	17.64 (87.48)	0.00 (0.00)	−17.64 (−49.81, 0)
Private acupuncturist	0.00 (0.00)	11.38 (86.66)	11.38 (0,43.28)
NHS X-rays	5.26 (15.77)	7.48 (34.01)	2.22 (−4.98, 15.51)
NHS US scans	2.04 (10.39)	4.66 (29.11)	2.61 (−3.37, 12.32)
NHS CT scans	0.00 (0.00)	1.55 (11.82)	1.55 (0, 5.53)
NHS blood tests	0.68 (3.03)	1.86 (8.19)	1.18 (−0.49, 3.99)
NHS MRI investigations	2.66 (19.37)	7.29 (41.11)	4.63 (−4.86, 19.45)
Resource use other			
NHS Hip-related surgery	237.55 (1211.03)	0.00 (0.00)	−237.54 (−629.5, 0)
‘Over-the-counter’ treatments	12.78 (31.73)	14.96 (38.27)	2.1 (−9.06, 16.38)
Total costs (complete cases)			
Total cost (NHS) (£)	385.29 (1331.90)	158.62 (212.96)	−226.67 (−727.37, 55.64)
Total costs (imputed)			
*n*	67	66	
Total cost (NHS) (£)	327.45 (1188.68)	165.85 (212.71)	−161.59 (−583.95, 54.18)

Values are mean (s.d.) unless stated otherwise.

a BCT+US-T minus BCT.

b Trial intervention cost including health professional and ultra sound machine cost. BCT: best current treatment; BCT+US-T: BCT plus US-guided injection of triamcinolone and lidocaine; ICER: incremental cost-effectiveness ratio; NHS: National Health Service.

### Sensitivity analysis

The direction of the base-case result did not change when total healthcare and societal costs were considered or when a complete-case analysis was undertaken (Tables 4 and 5). Similarly, the direction of the base-case result remained the same when responses from each time point were converted into index scores using the interim cross-walk value set (Supplementary Table S3, available at *Rheumatology* online). Sensitivity analysis from the societal perspective showed a societal benefit from fewer work days lost because of hip pain corresponding to a mean productivity cost saving of £450 for BCT+US-T compared with BCT alone (data available for 6-month follow-up; Tables 4 and 5). Similarly, BCT+US-T remained cost-effective (99% probability of being cost-effective) when the two surgery-related costs reported in the BCT group were excluded from the analysis.

**Table 5.** Total costs and cost-effectiveness analysis results, by intervention arm

Imputed analysis		BCT (*n* = 67)		BCT+US-T (*n* = 66)		
Total NHS cost (base case), £		327.45 (1188.68)		165.85 (212.71)		
Mean difference (95% CI), £		−161.59 (−583.95, 54.18)				
Total health care cost, £		398.69 (1194.63)		211.87 (256.72)		
Mean difference (95% CI), £		−186.82 (−569.02, 43.98)				
Total societal cost, £		604.48 (2191.65)		294.15 (391.23)		
Mean difference (95% CI), £		−310.33 (−1033.37, 79.96)				

	Cost-effectiveness outcomes over 6 months			Probability SC is cost-effective at cost-effectiveness threshold of		
		
	Mean incremental costs (BCT+US-T minus BCT) (95% CI), £	Mean incremental QALYs (BCT+US-T minus BCT) (95% CI)^a^	ICER	£20 000 per QALY	£30 000 per QALY	£50 000 per QALY

Base case						
NHS perspective	−161.59 (−583.95, 54.18)	0.05572 (0.0308, 0.0806)	Dominated^b^	0.99	0.99	0.99
Sensitivity analysis 1: alternative perspectives						
Healthcare perspective	−186.82 (−555.88, 47.01)	0.05572 (0.0308, 0.0806)	Dominated^b^	0.99	0.99	0.99
Societal perspective	−310.33 (−1040.92, 64.34)	0.05572 (0.0308, 0.0806)	Dominated^b^	0.99	0.99	0.99
Sensitivity analysis 2: complete-case analysis						
NHS cost perspective	−226.67 (−727.37, 55.64)	0.0562 (0.0258, 0.0864)	Dominated^b^	0.99	0.99	0.99
Sensitivity analysis 3: *post hoc* sensitivity analysis: NHS perspective excluding hip surgery						
NHS perspective	16.44 (−80.87, 93.59)	0. 0562 (0.0258, 0.0864)	£345 per QALY gained^c^	0.99	0.99	0.99

a Adjusted for baseline health-related quality of life.

b Mean ICER in south-east quadrant of the cost-effectiveness plane where intervention was less costly and more effective.

c Mean ICER in north-east quadrant of the cost-effectiveness plane where intervention was more expensive but more effective. BCT: best current treatment; BCT+US-T: BCT plus US-guided injection of triamcinolone and lidocaine; ICER: Incremental cost-effectiveness ratio; NHS: National Health Service; SC: Stratified care; QALY: quality-adjusted life year.

### Cost-utility analysis

**Figure 1.**
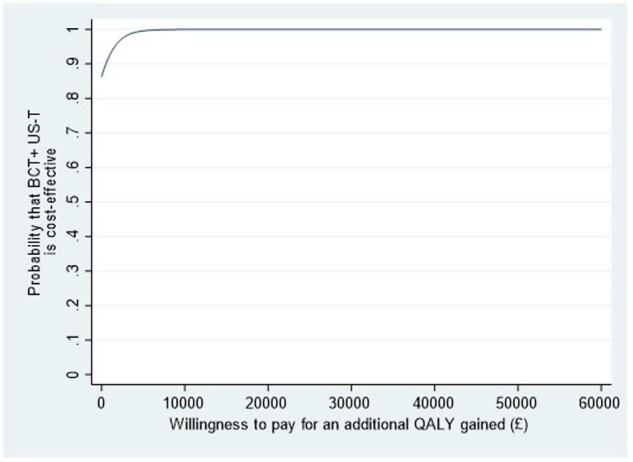
Cost-utility acceptability curve comparing USGI ultrasound-guided intra-articular hip injection combined with best current treatment (BCT+US-T) with best current treatment (BCT)

The base-case analysis showed that BCT+US-T was more effective and less costly than BCT (cost difference −£161.59; 95% CI: −£583.95, 54.18; QALY difference 0.0487; 95% CI: 0.0091, 0.0886), resulting in a position of dominance (Table 5). The dominance of the intervention was shown in a 99% probability of intra-articular corticosteroid injection for hip OA with best current treatment being cost-effective at a willingness to-pay threshold of £20 000 per QALY gained (Table 5, Fig. 1, and Supplementary Fig. S1 available at *Rheumatology* online).

## Discussion

### Summary of main findings

This study sought to assess the cost-effectiveness of adding an USGI of triamcinolone acetonide and lidocaine hydrochloride to best current care comprising advice/education and exercise in adults with hip OA [7]. Our results reveal that BCT+US-T was less costly (due to participants going on to have less additional healthcare use over 6 months) and more effective in terms of HRQOL than advice and education alone with significantly higher total QALYs at 6 months. The cost–utility showed that the BCT+US-T, was more cost-effective than BCT alone from an NHS, healthcare and societal perspective.

These findings complement the clinical effectiveness analysis which found greater pain reduction and improvement in function over 6 months in adults with hip OA with BCT+US-T than BCT alone [7]. A further finding was that days off work; NHS consultant, private physiotherapist and chiropractor appointments; and hip surgery (*n* = 2) were more common in the BCT-alone group over the 6-month follow-up period. However, BCT+US-T remained cost-effective when surgery costs were excluded in a *post hoc* sensitivity analysis. In the BCT-alone group, HRQOL was lower at 6 months’ follow-up compared with baseline. Patients in this group did not receive an injection, and therefore might have experienced resentful demoralization that accentuated these differences in outcomes at 6 months between the two groups.

A previous review identified RCTs of corticosteroid injection for hip OA compared with either local anaesthetic, saline or standard care and highlighted a lack of cost-effectiveness studies [5]. Recent guidelines from NICE identified no published economic evidence comparing intraarticular corticosteroid injections for managing OA at joints other than the knee [6].

### Strengths and weaknesses of the study

A major strength of this study is that it is the first RCT to report the cost-effectiveness of an ultrasound-guided intra-articular hip injection of triamcinolone acetonide and lidocaine hydrochloride compared to best current care comprising advice/education and exercise in adults with hip OA. Our study therefore considered a population where evidence of cost-effectiveness is lacking. The analysis was based on comprehensive resource use information reported from an NHS, healthcare and societal perspective including wider societal costs of work absence with good response rates. Furthermore, the analysis adopted robust health economic analysis approaches in line with standard methods. However, there were some limitations. The resource use and work-related data were primarily collected using a self-reporting approach which could potentially lead to inaccuracies particularly over longer periods of time [26]. However, the recall period was only 6 months. Nevertheless, this method is widely used in health economics and is a pragmatic approach to collecting resource use data in the absence of routine data sources. Moreover, any inaccuracies are likely to be balanced across the two groups.

This study must be considered in the context of the study setting. Our intervention was costed to be delivered mostly by physiotherapists with an extended scope injection skill, on the same day of assessment. Therefore, our findings may not be generalizable to other contexts where injections are administered under X-ray guidance, in operating theatres or by senior medical staff. Furthermore, injections are not routinely given on the same day as assessment, and waiting for treatment could incur further costs in the long term, which could potentially affect the cost–utility. Given that clinical pathways and administration vary from place to place, it would be useful to understand the value for money of different pathways for giving steroid hip injections to people with hip OA in the long term, taking into account method of guidance (e.g. ultrasound, X-ray guided), setting (e.g. community, out-patient, operating theatre), professional background of injectors (e.g. allied health professionals, nursing, medical) and accessibility (e.g. same day, waiting list).

In conclusion, this health economic analysis demonstrated that in community settings of musculoskeletal services, adding an ultrasound-guided intra-articular hip injection corticosteroid and local anaesthetic injection to one session of advice and education for people with hip OA is a cost-effective treatment compared with advice and education alone. These findings provide evidence to inform international guidelines and support decision making for policy makers, commissioners, general practitioners and clinicians in musculoskeletal services. Future research could explore the long-term cost-effectiveness of delivering hip injections to people with painful hip OA in different contexts and different clinical pathways.

## Supplementary Material

kead659_Supplementary_Data

## Data Availability

Data for this study will be made available to the scientific community on request after publication. Data will be made available for scientific purposes for researchers whose proposed use of the data has been approved by a publication committee. Data and documentation will be made available through a secure file exchange platform after approval of proposal and a data transfer agreement is signed (which defines obligations that the data requester must adhere to regarding privacy and data handling). Partially deidentified participant data limited to the data used for this work will be made available. For data access, please contact the corresponding author.
