# Supplementary material for: The cost-effectiveness of adding an ultrasound corticosteroid and local anaesthetic injection to advice and education for hip osteoarthritis
Source: Rheumatology (Oxford). 2023 Dec 12;64(1):165–72. doi: 10.1093/rheumatology/kead659 (PMC11701301; doi:10.1093/rheumatology/kead659)
Supplement: kead659_Supplementary_Data [file kead659_supplementary_data.docx]

**Supplementary table S1:** Unit Costs (£) (2018/2019 prices).

| Health care resource | Unit cost (£) | Unit Cost Source |
| --- | --- | --- |
| Primary care contacts: |  |  |
| General Practitioner: surgery consultation | 31 | PSSRU 2018 |
| Practice Nurse: surgery consultation | 36 | PSSRU 2018 |
| Hospital-based care |  |  |
| Consultant: Sciatica pain | 181 | DOH, 2018 |
| Physiotherapist | 55 | DOH, 2018 |
| Chiropractor | 55 | DOH, 2018 |
| Acupuncturist | 55 | DOH, 2018 |
| Osteopath | 55 | DOH, 2018 |
| Hospital nurse | 54 | DOH, 2018 |
| Private consultants | Costed to the NHS equivalent |  |
| Private other health care professionals | Costed to the NHS equivalent |  |
| Diagnostic tests: x-ray | 31 | DOH, 2018 |
| Diagnostic tests: CT scan | 90 | DOH, 2018 |
| Diagnostic tests: MRI scan | 141 | DOH, 2018 |
| Diagnostic tests: Blood test | 6 | DOH, 2018 |
| Hip Surgery | 6,295 | DOH, 2018 |
| Out-of-pocket treatments | Participant reported costs | *Not applicable* |
| Prescribed medication | Participant-specific | BNF, 2018 |
| Work absence/reduced productivity | Participant -specific | ONS, 2018 |

**Supplementary Table S2: Details of the unit costs and intervention costs per participant**

| Health care resource | Unit cost (£) | Unit cost source |
| --- | --- | --- |
| **HIT intervention** |  |  |
| Health Personnel |  |  |
| Rheumatologist | 66 | PSSRU 2018 |
| ESP/EPS Physio band 8a | 66 | PSSRU 2018 |
| Consultant Sonographer band 8b | 65 | PSSRU 2018 |
| Average Clinician unit cost (per 30 minutes) | 33.8 | PSSRU 2018 |
|  |  |  |
| Cost of US machine | 15,000 | Trial-reported costs |
| Expected life span | 10 years |  |
| Annuity factor, 10 years at 4% | 8.110 |  |
| Per patient cost for ultrasound machine | 0.7 |  |
|  |  |  |
| Total intervention cost per patient (Clinician + ultrasound cost) ^.^ | 33.54 | Trial-reported costs |
|  |  |  |

Supplementary Table S3: Descriptive and incremental health outcomes over 6 months for the base-case analysis and the complete case analyses. Values are mean (SD) scores unless stated otherwise.

|  | | BCT  n=67 | | BCT+ US-T  n=66 | | Difference^b^  (CI) (BCT+US-T – BCT) |
| --- | --- | --- | --- | --- | --- | --- |
| Primary (Imputed) EQ-5D analysis ^a^ | | | | | | |
| Baseline EQ-5D | 0.4971 (0.2174) | | 0.4915 (0.2365) | | -0.005  (-0.0852, 0.0696) | |
| 2 weeks EQ-5D | 0.4813 (0.2626) | | 0.6427 (0.2281) | | 0.1614  (0.0794, 0.2443) | |
| 2-month EQ-5D | 0.4486 (0.2784) | | 0.5855 (0.2577) | | 0.1369  (0.0464, 0.2292) | |
| 4-month EQ-5D | 0.4737 (0.2709) | | 0.5693 (0.2458) | | 0.0956  (0.0049, 0.1824) | |
| 6-month EQ-5D | 0.4928 (0.2387) | | 0.5004 (0.2403) | | 0.0075  (-.07603, 0.0871) | |
|  |  | |  | |  | |
| Unadjusted total QALYs | 0.2178(0.1101) | | 0.2638(0.1024) | | 0.0460  (0.0096, 0.0825) | |
| Adjusted total QALYs^c^ | - | | - | | 0.0477  (0.0257, 0.0699) | |
| Complete-case analysis | n=47 | | n=50 | |  | |
| Unadjusted total QALYs | 0.2224 (0.1154) | | 0.2748 (0.0925) | | 0.052  (0.0111, 0.9190) | |
| Adjusted total QALYs^c^. | 0.2165 | | 0.2662 | | 0.0477  (0.0209,0.0741) | |
|  |  | |  | |  | |

**Supplementary Figure S1**: Cost-utility plane comparing USGI ultrasound-guided intra-articular hip injection combined with best current treatment (BCT+US-T) with best current treatment (BCT).
